# Evaluating whether a peer-led dissonance-based eating disorder prevention program prevents onset of each eating disorder type

**DOI:** 10.1017/S0033291723000739

**Published:** 2023-04-11

**Authors:** Laura D’Adamo, Ata Ghaderi, Paul Rohde, Jeff M. Gau, Heather Shaw, Eric Stice

**Affiliations:** 1 Department of Psychiatry, Washington University School of Medicine, St. Louis, MO, USA;; 2 Department of Psychology and Center for Weight, Eating, and Lifestyle Science, Drexel University, Philadelphia, PA, USA;; 3 Division of Psychology, Department of Clinical Neuroscience, Karolinska Institutet, Stockholm, Sweden;; 4 Oregon Research Institute, Eugene, OR, USA; 5 Psychiatry and Behavioral Sciences, Stanford University School of Medicine, Stanford, CA, USA

**Keywords:** Dissonance, eating disorder, peer educators, prevention, thin-ideal internalization

## Abstract

**Background.:**

This study tested whether the dissonance-based *Body Project* eating disorder prevention program reduced onset of subthreshold/threshold anorexia nervosa (AN), bulimia nervosa (BN), binge eating disorder (BED), and purging disorder (PD) over long-term follow-up.

**Methods.:**

Data were combined from three prevention trials that targeted young women at high-risk for eating disorders (*N* = 1092; *M* age = 19.3). Participants were randomized to *Body Project* groups led by peer educators or expressive writing/educational controls and completed masked diagnostic interviews over 2- to 4-year follow-ups. Logistic regressions tested whether onset of each eating disorder over follow-up differed between *Body Project* and control participants.

**Results.:**

Peer-led *Body Project* groups produced a 46% reduction in onset of subthreshold/threshold BN and a 62% reduction in onset of PD relative to controls over follow-up. Rates of onset of subthreshold/threshold AN and BED did not significantly differ between peer-led *Body Project* participants and control participants.

**Conclusions.:**

Results support the dissemination of the peer-led *Body Project* for reducing future onset of BN and PD. This study and recent research suggest that thin-ideal internalization, the risk factor for eating disorders targeted in the *Body Project*, may be more relevant for predicting onset of BN and PD compared to AN and BED. Findings support the development of a version of the *Body Project* aimed to reduce risk factors that have predicted future onset of all four types of eating disorders (e.g. overvaluation of weight/shape, fear of weight gain), which may more effectively prevent all eating disorder types.

Eating disorders affect 13% of females and 6% of males and result in distress, functional impairment, and elevated morbidity (Allen, Byrne, Oddy, & Crosby, 2013; Stice, Marti, & Rohde, 2013a). Unfortunately, most individuals with eating disorders do not receive treatment (Swanson, Crow, Le Grange, Swendsen, & Merikangas, 2011). Thus, it is a public health priority to broadly implement evidence-based eating disorder prevention programs. To evaluate prevention effectiveness, trials should report effects on onset of both subthreshold and threshold eating disorders. Studies examining anorexia nervosa (AN), bulimia nervosa (BN), binge eating disorder (BED), as well as subthreshold cases of these eating disorders and purging disorder (PD), classified as Other Specified Feeding and Eating Disorders (OSFED) per DSM-5 (American Psychiatric Association, 2013), indicate that adolescent girls with both threshold and subthreshold eating disorders experience functional impairment, distress, suicidality, unhealthy body weight, and mental health services utilization (Allen et al., 2013; Smink, Van Hoeken, & Hoek, 2012; Stice et al., 2013a). Individuals with threshold *v*. subthreshold BN and BED have similar functional impairment, distress, and treatment care (Stice, Marti, Shaw, & Jaconis, 2009). Moreover, approximately 30% of those with subthreshold eating disorders later develop threshold eating disorders (Glazer et al., 2019), and one-third of those who seek treatment for an eating disorder have OSFED (Eddy, Doyle, Hoste, Herzog, & Le Grange, 2008; Stice et al., 2009).

Three prevention programs have significantly reduced eating disorder symptoms and onset of eating disorders (Ghaderi, Stice, Andersson, Enö Persson, & Allzén, 2020; Martinsen et al., 2014; Stice, Marti, Spoor, Presnell, & Shaw, 2008; Stice, Rohde, Shaw, & Gau, 2020; Stice, Rohde, Shaw, & Marti, 2013b). However, the *Body Project*, a dissonance-based eating disorder prevention program, is the only prevention program that produced reductions in these outcomes in multiple efficacy trials conducted by independent teams compared to both minimal intervention control conditions and credible alternative interventions (Becker et al., 2010; Ghaderi et al., 2020; Halliwell & Diedrichs, 2014; Mitchell, Mazzeo, Rausch, & Cooke, 2007; Serdar et al., 2014; Stice et al., 2008, 2020). The *Body Project* is also the only prevention program that affects objective measures; it has reduced brain reward region responsivity to thin models (Stice, Yokum, & Waters, 2015b), positive implicit attitudes toward thin models (Kant, Wong-Chung, Evans, Stanton, & Boothroyd, 2019), attentional bias for thin models (Tobin, 2020), and ECG-assessed cardiac risk (Green et al., 2017). Additionally, it is the only prevention program to show efficacy across racial and ethnic groups, in numerous countries, for heterosexual and non-heterosexual females and males, and for a broad range of ages (e.g. AlShebali, Becker, Kellett, AlHadi, & Waller, 2021; Brown, Forney, Pinner, & Keel, 2017; Brown & Keel, 2015; Casasnovas *et al*. 2021; Rohde, Stice, Shaw, Gau, & Ohls, 2017; Rodriguez, Marchand, Ng, & Stice, 2008; Shaw, Rohde, Desjardins, & Stice, 2020; Stice, Onipede, Shaw, Rohde, & Gau, 2021b; Unikel-Santoncini, de León-Vázquez, Rivera-Márquez, Bojorquez-Chapela, & Méndez-Ríos, 2019).

The dual pathway model of bulimic-spectrum eating disorders (Stice & Van Ryzin, 2018) posits that pressure for thinness and pursuing the thin beauty ideal contribute to body dissatisfaction, increasing risk for dietary restriction and negative affect, which increase risk for binge eating and compensatory weight control behaviors. This model has garnered prospective support with clinically meaningful effect sizes in numerous studies (Allen, Byrne, & McLean, 2012; Fairburn, Cooper, Doll, & Davies, 2005; Favaro, Ferrara, & Santonastaso, 2003; Stice & Van Ryzin, 2018). The *Body Project* aims to reduce pursuit of the thin ideal among individuals at high-risk for eating disorders. In *Body Project* groups, participants critique the thin ideal in verbal, written, and behavioral exercises. These activities theoretically induce cognitive dissonance, prompting participants to reduce pursuit of the thin ideal to align their attitudes with their public behaviors. Reductions in pursuit of the thin ideal mediate effects of the intervention on eating disorder symptom reductions (Seidel, Presnell, & Rosenfield, 2009; Stice, Presnell, Gau, & Shaw, 2007). Additionally, high-dissonance versions of this program produce greater symptom reductions than low-dissonance versions (Green, Scott, Diyankova, Gasser, & Pederson, 2005; McMillan, Stice, & Rohde, 2011).

In a study examining risk factors that predicted future onset of each eating disorder type, pursuit of the thin ideal predicted future onset of BN, BED, and PD, but not future onset of AN (Stice, Gau, Rohde, & Shaw, 2017). Similar findings emerged when including data from an additional 680 participants (Stice, Desjardins, Rohde, & Shaw, 2021a). The fact that the *Body Project* focuses solely on reducing pursuit of the thin ideal suggests that it may more effectively prevent future onset of BN, BED, and PD than AN. To address this question, we combined data from three large randomized trials that used parallel recruitment and assessment procedures to test whether the *Body Project* reduces future onset of some eating disorders but not others. No report has tested whether an eating disorder prevention program prevented onset of each of the four types of eating disorders. We focused on *Body Project* groups that were implemented or co-implemented with peer educators because a meta-analytic review found that the *Body Project* has significantly reduced future onset of any eating disorder only when groups were led by peer educators (Stice, Onipede, & Marti, 2021c). Indeed, peer-led *Body Project* groups produced a significantly larger reduction in future onset of any eating disorder than clinician-led *Body Project* groups in a recent trial (Stice et al., 2020). Theoretically this is because health promotion interventions are perceived as more credible when delivered by individuals who are more like group participants (Cialdini, 2007). We combined data from peer-led *Body Project* groups that were implemented in-person and virtually, as virtual implementation of the *Body Project* has been shown to effectively prevent eating disorder onset over 24-month follow-up compared to expressive writing controls (Ghaderi et al., 2020). We hypothesized that participants assigned to peer-led *Body Project* groups will show significantly greater reductions in rates of future onset of BN, BED, and PD over follow-up relative to control participants, but not significantly greater reduction in AN onset.

## Method

### Participants and Procedures

We combined data from one efficacy trial [Trial 1 (completed prior to ClinicalTrials.gov); Stice et al., 2008]; one task-shifting implementation trial [Trial 2 (ClinicalTrials NCT01949649); Stice et al., 2020]; and a third trial that evaluated virtually implemented *Body Project* (*vBody Project*) groups (Trial 3 (ClinicalTrials NCT02567890); Ghaderi et al., 2020) resulting in a sample of 339 participants who completed peer-led *Body Project* groups (247 in-person, 92 virtual) and 753 control participants. In the total combined sample (*N* = 1092), the average age was 19.3 (S.D. = 5.36, range: 14–64). Average parental education in the total sample was 40% graduate degree, 31% college graduate, 15% some college, 10% high school graduate, 2% some high school, and 2% grade school graduate. In Trials 1 and 2, 66% of participants were White, 11% Asian, 8% Hispanic, 5% Black, 2% Native American, 1% Pacific Islander, and 7% multiracial. Trial 3 did not collect data on race and ethnicity. Data were collected in several regions of the United States (Trials 1 and 2) and Sweden (Trial 3), which may improve generalizability.

### Design of randomized prevention trials

Mailings and fliers recruited female participants for trials evaluating body acceptance interventions at high schools (Trials 1 and 3) and colleges (Trials 1 and 2). The sole inclusion criterion was that participants have body image concerns as indicated by a positive response to the question ‘Do you have body image concerns?’ (Trials 1 and 2) or ‘Are you dissatisfied with your body?’ (Trial 3). In Trial 3, all high schools in Sweden were asked to put up ads, psychology students were sent as project ambassadors to schools, and Facebook and Instagram ads were used. Participants who met criteria for DSM-IV (American Psychiatric Association, 2000) AN or BN were excluded from Trials 1 and 2; participants with BED or PD were not excluded from Trials 1 and 2 because these trials had started before publication of DSM-5 criteria and BED and PD were not included in DSM-IV. Trial 3 excluded those with a current eating disorder, severe depression, suicidality, or other serious conditions that required psychiatric care. For all three trials, informed consent was obtained from participants (and parents, if minors).

Trial 1 participants were randomized to the *Body Project* group intervention or an assessment-only control. Trial 2 participants were randomized to peer-led *Body Project* groups or an eating disorder education video control. Trial 3 participants were randomized to a virtually-delivered *Body Project* intervention or an expressive writing control. Trial 1 participants completed surveys and interviews at baseline, post-intervention, and at 6-, 12-, 24-, and 36-month follow-up; Trial 2 participants completed surveys and interviews at baseline, post-intervention, and at 6-, 12-, 24-, 36-, and 48-month follow-up; Trial 3 participants completed surveys and interviews at baseline, post-intervention, and at 6-, 12-, 18-, and 24- month follow-up. Additional details can be found in Stice et al. (2008, 2020) and Ghaderi et al. (2020).

### Interventions

#### Body Project

The *Body Project* consisted of 4 (3 sessions for Trial 1) weekly, one-hour group sessions with 5–9 participants. Participants voluntarily engaged in verbal, written, and behavioral exercises wherein they critiqued the thin ideal during the sessions and in home exercises. Intervention scripts are available at: http://www.bodyprojectsupport.org. In Trial 1, *Body Project* groups were led by the last author or graduate students and undergraduate co-facilitators (see Stice et al., 2008 for details on training and supervision). For Trial 2, we recruited peer educators from established peer educator programs on three college campus to conduct *Body Project* groups. Pairs of facilitators delivered the intervention. The acute effects report (Stice et al., 2017) provides greater detail regarding the selection criteria and demographics for leaders, and information regarding training and supervision. Trial 3 evaluated *Body Project* groups delivered virtually via Google Hangouts. Psychology undergraduates were trained as group leaders. Groups were run by one leader virtually via the Google Hangouts app and group members told they could participate anonymously if desired. Those randomized to the expressive writing condition received information on how to complete expressive writing exercises (see Ghaderi et al., 2020 for details).

### Control conditions

#### Expressive writing condition

The expressive writing condition consisted of brief written instructions sent to participants weekly over a one-month period, instructing participants to write about their thoughts, images, emotions, and whatever comes to their mind related to their body for 40 min (see Ghaderi et al., 2020 for details).

#### Educational video condition

Participants were asked to view *Dying to Be Thin* (WGBH Video, 2000), a documentary on eating disorders, body dissatisfaction, and body acceptance. Participants were sent a web page where they could view the video for free (see Stice et al., 2020 for details).

#### Educational brochure condition

Participants were mailed an educational brochure from the National Eating Disorders Association describing negative and positive body image, noting that negative body image is associated with increased risk for eating disorders, and offering 10 steps for achieving positive body image. Participants were also given a referral list of local mental health treatment providers with expertise in eating disorders at each assessment (see Stice et al., 2008).

### Measures

#### Eating disorder symptoms and diagnoses

Trials 1 and 2 used the semi-structured Eating Disorder Diagnostic Interview (EDDI) to assess DSM-IV eating disorder symptoms (these trials started before DSM-5 but collected data used to approximate DSM-5 criteria). Diagnostic interviews were conducted in person at baseline and over follow-up, unless the participant moved from the area or was unable to complete in-person interviews, in which case they were completed by phone. Frequency of binge eating, vomiting, laxative/diuretic use, fasting, and excessive exercise, and overvaluation of weight/shape, feeling fat, and fear of weight gain were assessed monthly over the intervals between assessments. Participants who endorsed binge eating were asked about distress regarding binge eating, rapid eating, eating until uncomfortably full, eating large quantities of food when not hungry, eating alone because of embarrassment, feeling disgusted, depressed, or guilty after overeating. We used the monthly data on eating disorder symptoms to determine when participants first met criteria for threshold/subthreshold eating disorders (operationalized in Stice et al., 2017). EDDI diagnoses have shown one-week test-retest reliability (κ = 0.79) and inter-rater agreement (κ = 0.75), and participants with *v*. without EDDI-diagnosed eating disorders show greater functional impairment, distress, and mental health treatment (Stice et al., 2008, 2013a, 2017).

Trial 3 used the Eating Disorders Examination (EDE) to assess DSM-5 diagnoses of eating disorders via phone. The EDE (Fairburn & Cooper, 1993) consists of a substantial number of questions and can be supplemented by questions chosen by the interviewer. Only items used to diagnose DMS-5 AN, BN, BED, and OSFED were administered, which included subthreshold levels of AN, BN, and BED, as well as PD. The time frame of the EDE was adapted at the 6, 12, and 24-month follow-up time points to capture the entire period since the previous assessment.

Diagnoses were conferred using the monthly symptom data collected in interviews. Diagnostic criteria for threshold and subthreshold eating disorders used in this study, which approximate but are not identical to DSM-5 criteria, are in Table 1.

### Statistical methods

Logistic regression models tested whether the onset of each subthreshold or threshold eating disorder over follow-up was significantly lower for in-person or virtual *Body Project* conditions *v*. expressive writing or educational video/brochure control conditions among participants free of the eating disorder of interest at baseline. Separate models were estimated for AN, BN, BED, and PD. All models included intervention condition and sets of dummy-coded variables which adjusted for all differences between studies (i.e. type of control group, program delivery, number of *Body Project* sessions, length of follow-up, and instrument used to assess eating disorder symptoms and diagnosis) as fixed effects.

Rates of onset of AN in this study were relatively low compared to the other eating disorders. Thus, we also conducted equivalence tests using the R package TOSTER (Version 0.3) and two one-sided test procedures (TOTS; Lakens, Scheel, & Isager, 2018) to evaluate whether observed group differences in onset of AN during follow-up were significantly larger than the smallest effect size of interest (SESOI). We set the SESOI for AN at an incidence difference of 10% between conditions, below a threshold at which broad implementation of a prevention program would not be warranted. We used a one-tailed *p* value of 0.05 for inferential tests because we were testing a directional hypothesis and because this maximized power, which is the approach we have used in our previous reports that tested for a reduction in onset of any eating disorder in our prevention trials and has been used in prior clinical research (Dziuk et al., 1998; Fry, Pine, Jones, & Meimban, 2011; Glei, Goldman, Lin, & Weinstein, 2012). Importantly, all past reports from our trials showed lower rates of future onset of any eating disorder in *Body Project v*. control conditions (Ghaderi et al., 2020; Stice et al., 2008, 2020). Preliminary analyses included comparisons of future onset of any eating disorder for in-person *v*. virtual Body Project participants, and for educational video/brochure *v*. expressive writing control participants.

## Results

### Preliminary analyses

In-person *Body Project* participants did not differ in onset of subthreshold/threshold BN compared to virtual *Body Project* participants (3.7% *v*. 1.1%; OR 3.49, *p* = 0.239) or in rates of onset of subthreshold/threshold BED (5.7% *v*. 2.2%; OR 2.73, *p* = 0.188). In-person *Body Project* participants had greater onset of subthreshold/threshold AN and subthreshold/threshold PD compared to virtual *Body Project* participants over follow-up (1.2% *v*. 0.0% and 2.9% *v*. 0.0%, respectively). However, due to small and zero onset rates of future subthreshold/threshold AN and PD, reliable estimates of odds ratios were not possible. The differences in onset rates of subthreshold/threshold AN and PD for in-person *v*. virtual *Body Project* groups may be driven in part because the trials that evaluated in-person *Body Project* groups had a 3- and 4-year follow-up, whereas the trial that evaluated virtual *Body Project* groups had only a 2-year follow-up. Overall, analyses supported combining participants from in-person and virtual *Body Project* groups into a single active prevention group.

Educational video/brochure control participants did not differ in onset of subthreshold/threshold BN compared to expressive writing controls (6.2% *v*. 1.9%; OR 3.48, *p* = 0.089), subthreshold/threshold BED (5.3% *v*. 4.7%; OR 1.14, *p* = 0.788), or PD (3.7% *v*. 5.8%; OR 1.58, *p* = 0.398) over follow-up. Educational video/brochure control participants had lower rates of subthreshold/threshold AN compared to expressive writing controls (0.0% *v*. 1.4%). However, due to small and zero onset rates of future subthreshold/threshold AN, reliable estimates of odds ratios were not possible. Again, analyses supported combining participants from various control conditions into a single comparison group.

### Onset of specific eating disorders

Table 2 details onset over 2- to 4-year follow-up for each eating disorder in the two conditions. Among peer-led *Body Project* participants without any eating disorder at baseline, eating disorder onset over follow-up was 10.6% (*n* = 36). Over follow-up, 3 peer-led *Body Project* participants developed subthreshold/threshold AN (0.9%), 10 developed subthreshold/threshold BN (3.0%), 16 developed subthreshold/threshold BED (4.8%) and 7 developed subthreshold/threshold PD (2.1%). Among participants in the control conditions without eating disorders at baseline, eating disorder onset over follow-up was 17.4% (*n* = 131). In total, 9 control participants developed subthreshold/threshold AN (1.2%), 42 developed subthreshold/threshold BN (5.6%), 39 developed subthreshold/threshold BED (5.2%) and 41 developed subthreshold/threshold PD (5.5%) over follow-up. Reductions in eating disorders for peer-led *Body Project* participants relative to control participants was 25% for subthreshold/threshold AN, 46% for subthreshold/threshold BN, 8% for subthreshold/threshold BED, and 62% for subthreshold/threshold PD.

Logistic regression models that tested whether study condition predicted onset of each type of eating disorder found a reduction in onset of subthreshold/threshold AN among peer-led *Body Project* participants *v*. control participants over the follow-up period which did not reach significance (OR 0.56, *p* = 0.213). Results of the equivalence tests were statistically significant (*z* = 15.05, *p* < 0.001), thus we accepted the alternative hypothesis and concluded that differences in onset of AN between *Body Project* participants and control participants were smaller than what we consider meaningful. A statistically significant reduction in onset of subthreshold/threshold BN among peer-led *Body Project* participants *v*. control participants over the follow-up period emerged (OR 0.47, *p* = 0.0285). A reduction in subthreshold/threshold BED among peer-led *Body Project* participants *v*. control participants over the follow-up period emerged but did not reach significance (OR 0.70, *p* = 0.149). A statistically significant reduction in onset of subthreshold/threshold PD for peer-led *Body Project* participants compared to control participants over follow-up was also observed (OR 0.35, *p* = 0.009).

Of note, we conducted Cox proportional hazard models and mixed-effects hazard models (including a random intercept per participant) as sensitivity analyses. Results of these models were consistent with results of the logistic models reported above. We also conducted post-hoc two-tailed logistic models and found that *Body Project* had significantly lower rates of onset of subthreshold/threshold PD in those models (*p* = 0.016); the differences in onset rates of BN were not significant (*p* = 0.065) in the two-tailed models.

## Discussion

This study evaluated whether participants in the peer-led *Body Project* differed in rates of onset of each of the four primary DSM-5 eating disorders (AN, BN, BED, and PD) compared to control conditions in a combined sample from three large eating disorder prevention trials. Results indicated that peer-led *Body Project* groups produced a statistically significant 46% reduction in onset of subthreshold/threshold BN and a statistically significant 62% reduction in onset of PD relative to controls during the follow-up period. These prevention effects are critical, given that <50% of individuals who experience BN or PD achieve full recovery (Forney, Crosby, Brown, Klein, & Keel, 2021; Steinhausen & Weber, 2009). In addition to high rates of comorbidity and physical consequences, BN has been independently associated with suicidality above and beyond risk predicted by comorbid disorders (Bodell, Joiner, & Keel, 2013). Although advances have been made in the study of PD since its addition to DSM-5 as an OSFED (American Psychiatric Association, 2013), no published randomized trials evaluating the treatment of PD exist (Keel, 2019). No other prevention program has been shown to reduce onset of these two types of eating disorders. Thus, the *Body Project* holds considerable promise for reducing the population prevalence of these disorders if broadly implemented.

BN and PD have similar risk profiles, as several shared risk factors (i.e. thin-ideal internalization, body dissatisfaction, dietary restraint, negative affect, psychosocial impairment) and prodromal symptoms (i.e. binge eating, compensatory behaviors, weight/shape overvaluation, fear of weight gain, feeling fat) have predicted the future onset of both disorders (Stice et al., 2021a). Additionally, individuals with both subthreshold/threshold BN and PD have been shown to develop compensatory weight control behaviors before binge eating, compared to the reverse temporal sequence of symptom emergence seen in BED, and tend to develop cognitive symptoms in tandem with compensatory behaviors (Stice et al., 2021a). Theoretically, cognitive symptoms (e.g. pursuit of the thin ideal) may drive use of compensatory behaviors, leading to binge eating onset. A key implication is that eating disorders emerge primarily because of thin-ideal internalization and other cognitive symptoms, which putatively drive compensatory weight control behaviors. This is consistent with the dual-pathway model, which argues that pursuing the thin ideal is key in developing eating disorders characterized by binge eating and compensatory weight control behaviors (Stice & Van Ryzin, 2018). An 8-year prospective test of the temporal sequencing of risk factor emergence hypothesized by the dual-pathway model supported this theory, concluding that prevention programs that effectively reduce pursuit of the thin ideal should reduce onset of bulimic-spectrum eating disorders (Stice & Van Ryzin, 2018). Results from the current study support this hypothesis, suggesting that the *Body Project* reduces pursuit of the thin ideal, in turn preventing the development of symptoms that characterize subthreshold/threshold BN and PD.

No significant difference in rates of onset of subthreshold/threshold AN emerged between peer-led *Body Project* and control participants. This outcome supports findings from a prospective study examining risk factors that predict future onset of eating disorders, which found that thin-ideal internalization did not predict onset of subthreshold/threshold AN (Stice et al., 2021a). These findings suggest that AN has a distinct risk profile compared to eating disorders in the binge eating/purging spectrum, and that prevention programs aimed to reduce pursuit of the thin ideal may not reduce the onset of subthreshold/threshold AN. However, this result should be interpreted with caution due to lower power caused by low onset rates of AN in our sample. The nonsignificant 25% reduction in future onset of subthreshold/threshold AN observed over follow-up may have reached significance in a larger sample and may still be clinically meaningful. Future research should identify strategies to recruit individuals at elevated risk for AN into eating disorder prevention trials to further investigate the effectiveness of the *Body Project* at preventing onset of AN.

The 8% reduction in subthreshold/threshold BED among *Body Project* participants *v*. combined control participants over follow-up did not reach significance. Given that pursuit of the thin ideal has been found to be a risk factor for BED onset in young women (Stice et al., 2021a), we expected a prevention program aimed to target this mechanism to reduce BED onset. However, growing evidence suggests that pursuit of the thin ideal may not be as salient of a risk factor for future BED onset as other risk factors. A 2017 study examining the prospective effects of cognitive risk factors on eating disorder onset found that, although the predictive effect of thin-ideal internalization on risk of future BED onset was significant, the effect was smaller than the effects of thin-ideal internalization on risk of future onset of BN and PD (Stice et al., 2017). This pattern of findings advances risk factor models for BED by suggesting that thin-ideal internalization may not be the main risk factor that contributes to BED onset and appears to be less relevant for predicting BED onset relative to BN and PD. A more recent study found that the effect sizes for the relations between thin-ideal internalization and onset of BN, PD, and BED (BN: SRD = 0.22; BED: SRD = 0.16; PD: SRD = 0.21) were smaller than the effect sizes for the predictive effects of overvaluation of weight/shape (BN: SRD = 0.42; BED: SRD = 0.36; PD: SRD = 0.44) and fear of weight gain (BN: SRD = 0.36; BED: SRD = 0.24; PD: SRD = 0.40; Stice et al., 2021a). The fact that overvaluation of weight/shape and fear of weight gain have predicted future onset of all four types of eating disorders and the effects were nearly twice as large for these risk factors *v*. pursuit of the thin ideal (Stice et al., 2021a) suggests that it would be useful to create a dissonance-based prevention program focused on reducing overvaluation of weight/shape and fear of weight gain, which may produce larger and more transdiagnostic eating disorder prevention effects. In post-hoc analyses for this study, we examined baseline to follow-up change in overvaluation of weight/shape from the diagnostic interviews and found no significant differences between *Body Project* and control participants (*p* = 0.779, *d* = 0.02), indicating that a refined version of the intervention should target this risk factor more directly.

Several limitations of this study should be considered. First, differences in the design of the trials that formed the current data-set (e.g. differences in control conditions and follow-up periods) likely introduced excess noise into the data. Second, the rate of onset of each of the four types of eating disorders, particularly AN, was relatively low, which reduced power. Consistent with past research of dichotomous outcomes having low incidence rates, we conducted one-tailed tests given no previous suggestion that prevention programs had iatrogenic effects. Confidence in the present results was enhanced by the secondary analyses exploring time to disorder onset, which replicated the pattern of onset effects. Third, this study involved only females, so results may not generalize to males or those who do not identify as either. Future directions include testing whether eating disorder prevention programs produce equal effects across gender and socioeconomic status. Fourth, Trials 1 and 2 occurred prior to the creation of DSM-5 but collected data that allowed us to approximate DSM-5 criteria and examine threshold and subthreshold diagnoses.

Findings indicated that the peer-led *Body Project* more effectively reduces onset of subthreshold/threshold BN and PD than onset of AN and BED. Although these results are promising, they highlight the need for prevention programs to better target transdiagnostic risk factors that have been found to predict future onset of all four types of eating disorders. Given that research has demonstrated that overvaluation of shape/weight and fear of weight gain emerged as predictors of incidence of all four types of eating disorders (Stice et al., 2021a), results support developing a refined version of the *Body Project* focused on reducing these two attitudinal risk factors, which have not yet been targeted in a prevention program.

## Figures and Tables

**Table 1.** Diagnostic criteria for threshold and subthreshold eating disorders

Subthreshold anorexia nervosa	BMI of between 90% and 85% of that expected for age and gender Definite fear of weight gain more than 25% of the days for at least 3 months Weight and shape were definitely an aspect of self-evaluation Missed one period in a three month period (unless on birth control)
Threshold anorexia nervosa	BMI of less than 85% of that expected for age and gender Definite fear of weight gain more than 50% of the days for at least 3 months Weight and shape one of the main aspects of self-evaluation Missing menstrual cycles in a three month period (unless on birth control)
Subthreshold bulimia nervosa	At least 2 uncontrollable binge eating episodes per month for at least 3 months At least 2 compensatory behavior episodes (i.e. self-induced vomiting, laxatives use, diuretic use, fasting, and excessive exercise to compensate for overeating) per month for at least 3 months Weight and shape was definitely an aspect of self-evaluation
Threshold bulimia nervosa	At least 8 uncontrollable binge eating episodes per month for at least 3 months At least 8 compensatory behavior episodes per month for at least 3 months Weight and shape was definitely one of the main aspects of self-evaluation
Subthreshold binge eating disorder	At least 2 uncontrollable binge eating episodes/days per month for at least 6 months Less than 1 compensatory behaviors on average per month during this period Marked distress about binge eating Binge eating was characterized by 3 or more of the following: rapid eating, eating until uncomfortably full, eating large amounts when not physically hungry, eating alone because of embarrassment, feeling disgusted, depressed, or guilty after overeating
Threshold binge eating disorder	At least 8 uncontrollable binge eating episodes/days per month for at least 6 months Less than 1 compensatory behaviors on average per month during this period Marked distress about binge eating Binge eating was characterized by 3 or more of the following; rapid eating, eating until uncomfortably full, eating large amounts when not physically hungry, eating alone because of embarrassment, feeling disgusted, depressed, or guilty after overeating
Purging disorder	At least 8 episodes of self-induced vomiting or diuretic/laxative use for weight control purposes per month for at least 3 months Less than 1 uncontrollable binge eating episode on average per month during this period Weight and shape was definitely an aspect of self-evaluation

**Table 2.** Incidence of onset over follow-up for each eating disorder in the two study conditions

	Control (*n* = 753)		*Body Project* (*n* = 339)	
Threshold or subthreshold	*n*	*%*	*n*	*%*
Anorexia nervosa	9	1.2	3	0.9
Bulimia nervosa	42	5.6	10	3.0
Binge eating disorder	39	5.2	16	4.8
Purging disorder	41	5.5	7	2.1
